# The History, Diagnosis and Treatment of Typhoid and of Typhus Fever; with an Essay on the Diagnosis of Bilious, Remittent and of Yellow Fever

**Published:** 1842-12

**Authors:** 


Art. V.—*The History, Diagnosis and Treatment of Typhoid and of Typhus Fever; with an Essay on the Diagnois of of Bilious Remittent and of Yellow Fever.* By Elisha Bartlett, M. D., Professor of the Theory and Practice of Medicine in Transylvania University. Philadelphia: Lea & Blanchard. 1842. pp. 393, 8vo.

This is a neat, unpretending volume, aiming, as the author expresses it, "at no other merit than that of being a methodical and compendious summary of the actual state of our knowledge upon two of the most common and most important diseases." Such a work supplies a want extensively felt by the the profession in this country. As justly remarked by Dr. Bartlett, many authorities on the subject of idiopathic fevers that were received as standard and classical, only a few years ago, are fast becoming obsolete. The treatises of Fordyce, Wilson Philip, Armstrong, Southwood Smith, and Tweedie, describe a form of fever rarely encountered by the American practitioner; and they are also very deficient in their pathology, as most of them are in their plans of treatment, which, however successful they may be in the disease as it appears in England, are not well adapted to the fevers of the United States. These works, in a word, are very far from representing the actual state of our knowledge upon the subject of fever. Other treatises on fever are either not in general circulation, or else are not well suited to the profession here, on account of the minuteness and complexity of their details.

Dr. Bartlett, without aiming at any thing original, has presented, in his treatise, a history and comparison of the two chief forms of fever, as they are now ascertained to exist, fuller and more discriminating than had been written. He has brought out clearly and strongly the means of diagnosis between the different species of fever; and in regard to the treatment of continued fevers, he has given a faithful account of the methods employed by the most eminent practitioners who have written on the subject. The full table of contents will enable the reader to consult any portion of the work without trouble, and on every head the details will be found accurate and clear, and, perhaps, minute enough for the student, and for the practitioner of medicine.

In his reference to the controversy concerning the contagious qualities of yellow fever, Dr. Bartlett has fallen into the common error of ranking Dr. Rush among the earliest advocates of the doctrine of the non-contagiousness of this disease. One of the most zealous and successful advocates of the doctrine of its domestic origin unquestionably he was; but he was a decided and unwavering contagionist until the year 1806 or 7, as his pupils of that period testify. About that time he surrendered the opinion that the fever was contagious, and announced the change of his views in the New York Medical Repository. These facts ought to be known, and it is due to historical accuracy that the position of Dr. Rush should be distinctly stated, as it is but just to Dr. Caldwell to admit, that he preceded Dr. Rush many years in avowing and defending the non-contagious nature of yellow fever. Dr. Caldwell, during a warm and protracted controversy on the subject, is believed to have been the only *public* and *writing* advocate of the doctrine in this country. We have his authority for saying, that he was supported in his opinion by the concurring belief of Dr. Physick, but this gentleman, as is well known, wrote but little on any subject, and never participated publicly in this dispute. Y.

